# Erythropoietin regulation of red blood cell production: from bench to bedside and back

**DOI:** 10.12688/f1000research.26648.1

**Published:** 2020-09-18

**Authors:** Senthil Velan Bhoopalan, Lily Jun-shen Huang, Mitchell J. Weiss

**Affiliations:** 1 Department of Hematology, St. Jude Children’s Research Hospital, 262 Danny Thomas Place, MS #355, Memphis, TN, 38105, USA; 2 Department of Cell Biology, University of Texas Southwestern Medical Center, 5323 Harry Hines Boulevard, Dallas, TX, 75390, USA

**Keywords:** Erythropoiesis, hypoxia-induced transcription factor, prolyl hydroxylase inhibitor, erythropoietin receptor signaling, JAK2, Janus Kinase 2

## Abstract

More than 50 years of efforts to identify the major cytokine responsible for red blood cell (RBC) production (erythropoiesis) led to the identification of erythropoietin (EPO) in 1977 and its receptor (EPOR) in 1989, followed by three decades of rich scientific discovery. We now know that an elaborate oxygen-sensing mechanism regulates the production of EPO, which in turn promotes the maturation and survival of erythroid progenitors. Engagement of the EPOR by EPO activates three interconnected signaling pathways that drive RBC production via diverse downstream effectors and simultaneously trigger negative feedback loops to suppress signaling activity. Together, the finely tuned mechanisms that drive endogenous EPO production and facilitate its downstream activities have evolved to maintain RBC levels in a narrow physiological range and to respond rapidly to erythropoietic stresses such as hypoxia or blood loss. Examination of these pathways has elucidated the genetics of numerous inherited and acquired disorders associated with deficient or excessive RBC production and generated valuable drugs to treat anemia, including recombinant human EPO and more recently the prolyl hydroxylase inhibitors, which act partly by stimulating endogenous EPO synthesis. Ongoing structure–function studies of the EPOR and its essential partner, tyrosine kinase JAK2, suggest that it may be possible to generate new “designer” drugs that control selected subsets of cytokine receptor activities for therapeutic manipulation of hematopoiesis and treatment of blood cancers.

## Introduction

Healthy human adults produce about 200 billion red blood cells (RBCs) daily to replace those lost by senescence. This process, termed erythropoiesis, is exquisitely regulated by an oxygen-sensing mechanism that has evolved to maintain RBC numbers within a narrow physiological range ^1– 3^. Central to this mechanism is erythropoietin (EPO), a cytokine secreted by the kidney in response to low blood oxygen tension. Circulating EPO binds its cognate receptor (EPOR) on bone marrow erythroid progenitors, triggering multiple signaling pathways that support differentiation into mature RBCs. Inherited and acquired abnormalities in EPO production, its downstream activities, or its regulation cause numerous human diseases associated with too many or too few RBCs. The same pathways have been shaped by evolution for adaptation to life under chronic hypoxia at high altitudes. Although much is known about the production of EPO and its biological activities after 40 years of research, the topic remains a rich source for biomedical discovery and therapeutics. This review focuses on recent insights into the oxygen-regulated production of EPO and its actions on post-natal bone marrow erythropoiesis. It is important to note that EPO–EPOR signaling also drives RBC production during embryogenesis through similar but distinct mechanisms ^4– 6^.

## History of EPO

In 1875, Denis Jourdanet and Paul Bert described anemia-like symptoms in patients living at high altitude and identified low blood oxygen level to be the primary mechanism ^7^. Building on this finding about 30 years later, Carnot and Deflandre discovered that infusion of serum from anemic rabbits into normal ones caused a rise in RBC count, predicting the existence of a circulating factor that stimulates erythropoiesis ^3^. In the early 1950s, studies using parabiotic rats validated the concept of a humoral erythropoiesis-stimulating agent ^8, 9^ that was shown by Erslev ^9^ to originate from the kidney ^10^. In 1977, Goldwasser’s group reported the purification of EPO from 2550 liters of urine collected from patients with aplastic anemia ^11^. Molecular cloning of the *EPO* gene in 1985 facilitated the manufacture of recombinant human EPO (rhEPO) protein for treating various forms of anemia ^12, 13^. This work led to discoveries of the EPOR by Lodish’s group in 1989 ^14^ and subsequently multiple downstream signaling pathways were characterized by many laboratories. An elaborate oxygen-sensing mechanism that regulates EPO production was discovered in the early 1990s by William Kaelin Jr., Sir Peter Ratcliffe, and Gregg Semenza, who received the 2019 Nobel Prize in Physiology or Medicine for this work ^15– 20^.

## Erythropoietic activities of EPO and EPOR

**Figure 1.**
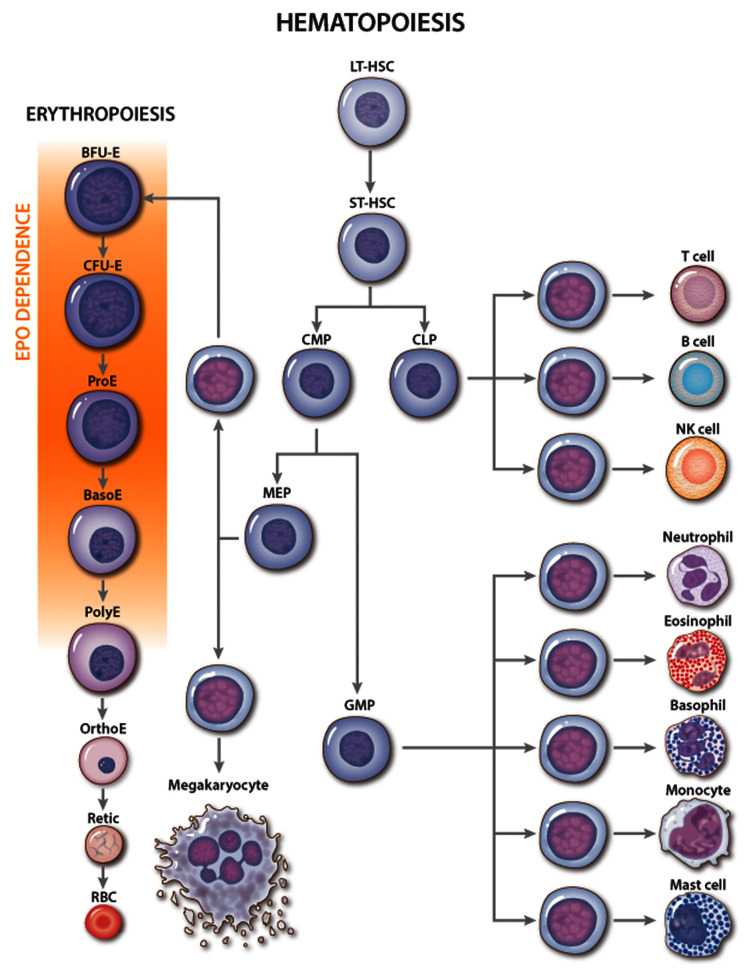
Erythropoietin (EPO) activity during erythropoiesis. Classic hierarchy of hematopoiesis with stages of red blood cell (RBC) development shown in greater detail. The major site of EPO action is indicated. Genetic and cell culture studies have shown that EPO is required for the development of CFU-E into late-stage erythroblasts. NK, natural killer. Multi-potent hematopoietic progenitors include the following: CLP, common lymphoid progenitor; CMP, common myeloid progenitor; LT-HSC, long-term engrafting hematopoietic stem cell; MEP, megakaryocytic-erythroid progenitor; ST-HSC, short-term hematopoietic stem cell. Committed erythroid progenitors include the following: BFU-E, burst-forming unit-erythroid; CFU-E, colony-forming unit-erythroid. Erythroid precursors include the following: BasoE, basophilic erythroblast; OrthoE, orthochromatic erythroblast; PolyE, polychromatic erythroblast; ProE, proerythroblast; Retic, reticulocyte.

Multi-potent hematopoietic stem cells undergo a series of differentiation steps that successively restrict developmental potential, giving rise to lineage-committed progenitors ( Figure 1) ^5^. The first identifiable erythroid progenitor, termed “burst-forming unit-erythroid” (BFU-E), is defined by its ability to generate large colonies with scattered clusters of erythroblasts in semi-solid medium. Differentiation of BFU-E produces “colony-forming units-erythroid” (CFU-E) that generate smaller colonies containing about 50 cells. Proerythroblasts, the first recognizable erythroid precursor, undergo further maturation steps, which include specialized cell divisions, reduced cell size, elimination of most organelles, development of a specialized cell membrane to facilitate microcirculatory transit, and accumulation of hemoglobin for oxygen transport ^1, 21, 22^. Terminal erythroid maturation occurs in bone marrow erythroblastic islands composed of erythroid precursors surrounding a central macrophage ^23^. The morphological and functional definitions of committed erythroid progenitors have been augmented by the identification of stage-specific cell surface markers ^24– 31^ and, more recently, the discovery of their transcriptional states using single-cell RNA sequencing (scRNAseq) ^32, 33^.

Although multiple cytokines support erythropoiesis ^34^, EPO is the key physiological regulator. Loss of EPO or derangements in EPO signaling in mice or humans cause anemia ^4, 35^ while excessive EPO production or EPOR signaling or both cause pathologically increased RBC numbers ^36– 38^. EPO acts mainly on CFU-E progenitors and proerythroblasts to maintain their survival and facilitate terminal maturation ( Figure 1) ^25, 39– 41^. Additionally, EPO can stimulate cell proliferation and drive multi-potent hematopoietic progenitors toward an erythroid fate ^40, 42^ but is not required for erythroid lineage commitment ^4^. *In vivo* administration of EPO leads to rapid skewing of multi-potential progenitors away from myeloid and toward the erythroid lineage and to altered gene expression in BFU-E and CFU-E progenitors ^32^.

## An oxygen-sensitive feedback loop regulates EPO production

**Figure 2.**
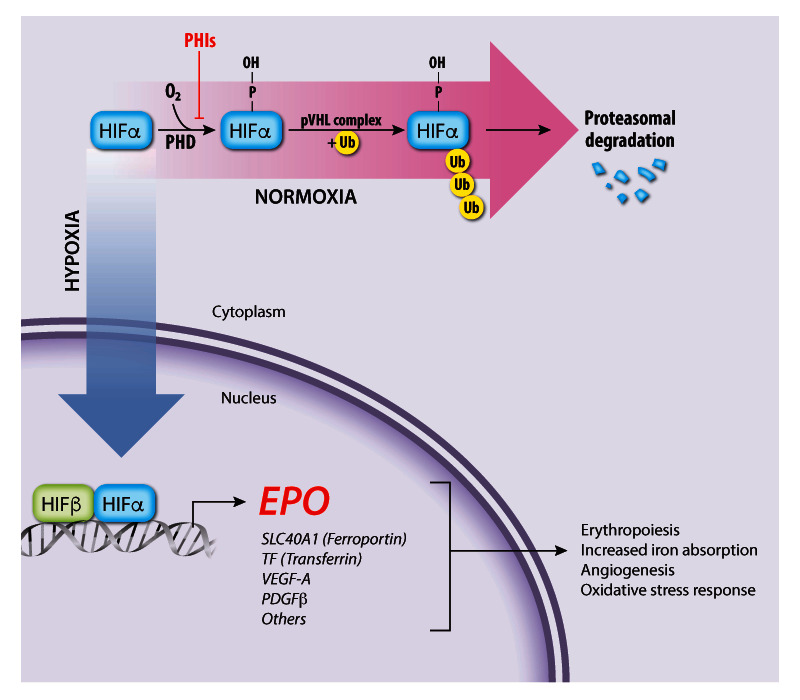
Regulation of endogenous erythropoietin (EPO) gene transcription by the oxygen-sensitive hypoxia-inducible factor (HIF) pathway The HIF transcription factor heterodimer (HIFα–HIFβ) activates the *EPO* gene and numerous other genes that promote tissue oxygen delivery. At high oxygen concentrations, prolyl hydroxylase (PHD) enzymes hydroxylate the HIFα subunit, targeting it for ubiquitination by the von Hippel–Lindau protein (pVHL) ubiquitin ligase complex followed by proteasomal degradation. Under hypoxia, PHD enzymes are inactive, thereby stabilizing HIF, which activates transcription of *EPO* and other target genes involved in tissue oxygen delivery. PHD inhibitors (PHIs) such as roxadustat and vadadustat stabilize HIFα and are under investigation for treating anemia associated with chronic renal failure. PDGFβ, platelet-derived growth factor beta; SLC40A1, solute carrier family 40 member 1; TF, Transferrin; VEGF-A, vascular endothelial growth factor A.

Post-natal EPO production occurs mainly in peritubular fibroblast-like interstitial cells of the kidney ^43– 50^ but also in liver, spleen, bone marrow, lungs, and brain ^51– 53^ and is regulated by blood oxygen levels through a transcriptional feedback loop ( Figure 2) ^15– 19^. The hypoxia-inducible transcription factor (HIF) complex binds hypoxia response elements in the *EPO* gene promoter to stimulate its transcription. Functional HIF is a heterodimer composed of an α subunit (HIFα) and a β subunit (HIFβ, also known as aryl hydrocarbon receptor nuclear translocator or ARNT). The stability of HIF is regulated by prolyl hydroxylase domain (PHD) enzymes, which use oxygen and 2-oxoglutarate to catalyze the hydroxylation of specific proline residues in HIFα, thereby stimulating binding of the HIF heterodimer to the von Hippel–Lindau protein (pVHL) component of an E3 ubiquitin ligase complex ^3, 54, 55^. Subsequent polyubiquitination of HIF leads to its proteasomal degradation. At low cellular oxygen concentrations, the PHD proteins are inactive and HIF is stabilized for target gene activation. Another 2-oxoglutarate–dependent oxygenase, factor inhibiting HIF (FIH), stimulates the oxygen-dependent hydroxylation of a specific asparagine residue in HIFα, which inhibits its activity by blocking HIFα binding to the transcriptional co-activator p300 ^55, 56^. In these ways, the PHD and FIH enzymes act as oxygen sensors that inhibit the production of EPO and other HIF targets under oxygen-replete conditions. Remarkably, HIF also activates hundreds of genes besides *EPO*. Other HIF target genes encode glycolytic enzymes, angiogenic factors, and iron uptake proteins, representing a concerted hypoxia response to increase RBC production, manufacture hemoglobin, enhance tissue perfusion, and promote oxygen-independent metabolism through glycolysis ^54, 57– 59^.

Mammals express three HIFα isoforms (HIF-1α, -2α, and -3α) and three PHD isoforms (PHD1, 2, and 3), each encoded by separate genes with overlapping but distinct tissue distributions and functions ^60^. The production of EPO in adult life is regulated mainly by HIF-2α and PHD2 ^61^. Perhaps not surprisingly, germline and somatic mutations affecting the PHD–HIF–EPO regulatory pathway are associated with erythrocytosis, anemia, abnormal angiogenesis, and cancer ^62, 63^. In mice and humans, loss-of-function mutations in *PHD2* and *VHL* or gain-of-function missense mutations that stabilize HIF-2α by inhibiting its binding to PHD2 or VHL cause erythrocytosis ^64^. An interesting gain-of-function mutation in the *EPO* gene (c.32delG) was recently identified to cause autosomal dominant erythrocytosis in a multi-generational pedigree ^65^. The single-nucleotide deletion introduces a frameshift into the main *EPO* mRNA but initiates excess production of EPO from what is normally a non-coding *EPO* mRNA transcribed from an alternative promoter in intron 1. Variants in the PHD–HIF–EPO pathway have also been selected for in evolution as an adaptive mechanism to living at high altitude. Some of these variants attenuate hypoxia-induced erythrocytosis that can cause deleterious hyperviscosity syndromes ^64, 66– 69^. These clinical observations highlight the exquisite and complex genetic regulation of EPO production and erythropoiesis. Of note, only one isoform of FIH has been identified. Ablation of the corresponding gene in mice causes metabolic alterations but does not appear to alter the canonical HIF functions in erythropoiesis or angiogenesis ^70^.

## EPO activities are mediated through the EPOR

**Figure 3.**
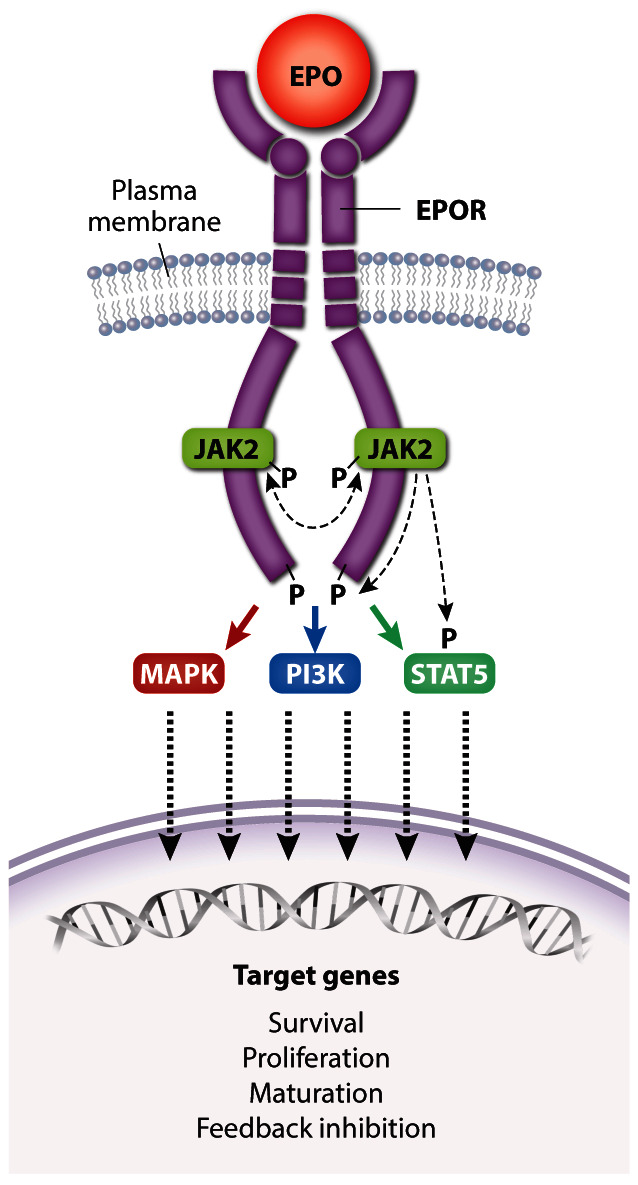
Activation of erythropoietin receptor (EPOR) by EPO. A single EPO molecule binds and stabilizes EPOR–JAK2 complex dimers, inducing a conformational change that initiates JAK2 trans-phosphorylation and activation. Active JAK2 phosphorylates multiple tyrosine residues on STAT5 and the cytoplasmic domain of EPOR, triggering a signaling cascade that activates numerous effector pathways contributing to biological activity. Dashed lines represent kinase activity. For simplicity, the kinase activity of only one JAK2 protein is indicated. Major signaling pathways activated by EPOR include Ras/MAPK, STAT5, and PI3K/Akt, which drive the expression of genes that promote erythroid progenitor survival, proliferation, and differentiation as well as feedback inhibition of EPOR signaling. MAPK, mitogen-activated protein kinase; PI3K, phosphoinositide-3 kinase; STAT5, signal transducer and activator of transcription 5.

EPO drives erythropoiesis by stimulating the EPOR on the surface of erythroid progenitors. The EPOR is a member of the type I cytokine receptor family distinguished by a conserved extracellular WSXWS amino acid motif, a single-transmembrane domain, and a cytoplasmic tail that lacks intrinsic tyrosine kinase activity ^71^. The proximal cytoplasmic domain of EPOR is bound by the JAK2 tyrosine kinase. Binding of a single EPO molecule to two EPOR molecules triggers a conformational change that stimulates JAK2 to initiate a multi-tiered signaling cascade ( Figure 3) ^72, 73^. Activated JAK2 phosphorylates itself and several tyrosine residues on the EPOR cytoplasmic tail, which serve as docking sites to engage SH2-containing signaling molecules such as the STAT5 (signal transducer and activator of transcription 5) transcription factor. Following phosphorylation and activation by JAK2, STAT5 enters the nucleus to activate numerous target genes ^74^. Biologically important erythroid STAT5 target genes include the following: *BCL2L1*, which prevents apoptosis of late-stage erythroblasts ^75– 77^; *ID1*, which promotes erythroblast expansion and survival ^78^; *TRIB3*, which regulates erythroid maturation ^79^; *SPI2A*, which encodes a serpin protease with antioxidant activities ^80^; and *TFRC* (transferrin receptor protein 1), which mediates iron uptake ^81, 82^. The recently discovered STAT5 target gene erythroferrone ( *ERFE*) encodes a hormone that acts on hepatocytes to inhibit their production of hepcidin, a different hormone that blocks intestinal iron absorption and release of iron stores from macrophage ^83^. By stimulating the production of ERFE in erythroblasts, EPO increases bioavailable iron for hemoglobin synthesis ^83, 84^. In addition to STAT5, EPOR activates the canonical Ras/mitogen-activated protein kinase (MAPK) and phosphoinositide-3 kinase (PI3K)/Akt pathways to enhance erythroid progenitor survival, proliferation, and differentiation ^3, 85– 89^. The Akt kinase also activates FOXO3, a transcription factor that induces genes that control antioxidant pathways ^90, 91^, cell polarity, and enucleation ^92^. Other signaling molecules activated by EPOR include Lyn kinase and PLCγ, although their contributions to erythropoiesis are less clear ^93, 94^.

Although some EPO–EPOR effectors can be linked directly to activation of a single linear signaling pathway, overgeneralizing this concept may be biologically inaccurate. As postulated for cytokine receptor signaling in general ^95^, the biological functions of EPOR are likely to be regulated by cross-communications between its numerous downstream signaling pathways and signaling by other cytokine receptors. In regard to the latter, cooperative signaling between the EPOR and stem cell factor receptor (KIT) is believed to promote erythropoiesis ^96– 99^. The EPOR also binds the type 2 transferrin receptor (TFR2), which is expressed in hepatocytes and erythroid progenitors. In hepatocytes, the TFR2 stimulates hepcidin production and germline *TFR2* mutations cause iron overload (hemochromatosis type 3) ^100^. In erythroid progenitors, TFR2 binds EPOR in the endoplasmic reticulum and facilitates its transport to the cell surface ^101^. The effects of TFR2 in erythroid progenitors appear to be context-dependent and are not fully resolved. In cultured erythroblasts, suppression of TFR2 inhibits erythropoiesis ^101^. In contrast, hematopoietic-specific ablation of the *Tfr2* gene in mice enhances erythropoiesis, likely by modulating EPO sensitivity ^102^. Expression of TFR2 in the kidney may inhibit EPO production ^103^. Interaction with iron-bound transferrin stabilizes TFR2 at the cell surface ^104^, representing a potential mechanism by which TFR2 coordinates erythropoietic rate and enteral iron uptake with circulating iron level.

Signaling through EPO–EPOR promotes both basal erythropoiesis, which maintains homeostasis by replacing erythrocytes lost by normal senescence, and “stress erythropoiesis” associated with increased synthetic demands caused by bleeding, excessive RBC destruction, or hypoxia. Relatively low concentrations of EPO during basal erythropoiesis are thought to act mainly by inhibiting apoptosis of erythroid progenitors, while stress erythropoiesis induces higher EPO concentrations that can drive hematopoietic differentiation toward the erythroid fate ^25, 32, 40, 41^. In line with this notion, EPO is able to act like a dimmer switch in activating STAT5 over a wide concentration range ^105^, and functional genomic approaches are beginning to identify direct targets of EPO-activated STAT5 in erythropoiesis ^74^. Different EPO concentrations during basal and stress erythropoiesis are likely to engage distinct signaling modalities, as revealed by a “knock-in” mouse strain in which EPOR is replaced with a truncated version (EPOR-HM) that binds and activates JAK2 but lacks the cytoplasmic portion containing all JAK2 tyrosine substrates ^106^. EPOR-HM mice are viable with a mild defect in steady-state erythropoiesis but are unable to support stress erythropoiesis ^106^. Thus, phosphotyrosine signaling from the EPOR is selectively required for stress erythropoiesis. The signaling pathways that are activated in response to high EPO concentrations differ depending on whether stress is chronic or acute. For example, STAT5-mediated activation of *BCL2L1* occurs rapidly after acute bleeding or hypoxia, and then decays, even if high levels of EPO persist. In contrast, persistent or chronic stress conditions such as β-thalassemia elicit a distinct set of EPOR signaling pathways that include the EPOR-mediated suppression of pro-apoptosis genes *FAS* and *BCL2L11* (formerly *BIM*) ^32^.

## EPO signal termination

Activation of EPOR by EPO is balanced by complex negative feedback mechanisms that fine-tune and inhibit signaling to prevent excessive RBC production. Initial evidence for this came from studies of a Finnish family ascertained through an Olympic cross-country skier ^107^. This family and others discovered subsequently were found to have erythrocytosis caused by EPOR-truncating mutations that eliminate portions of the cytoplasmic domain, which later was found to negatively regulate EPOR signaling by recruiting various inhibitory proteins, including the tyrosine phosphatase PTPN6, members of the suppressor of cytokine signaling (SOCS) protein family, SH2B adapter protein 3 (SH2B3, LNK), and the p85 regulatory subunit of PI3K ^38^. Mechanistically, PTPN6 attenuates EPOR signaling by dephosphorylating JAK2 ^108, 109^. CISH and SOCS3 block access of STAT5 to the EPOR, whereas SOCS1 binds to the JAK2 kinase domain and reduces its tyrosine kinase activity ^110^. Transcription of SOCS1, SOCS3, and CISH are induced by STAT5, forming a negative feedback loop ^111^. Mutations in JAK2 at the SOCS3 binding site and mutations in SOCS3 occur in patients with erythrocytosis ^112, 113^. The SH2B3 protein (LNK) is upregulated and phosphorylated in response to EPO and inhibits EPOR signaling by binding phosphotyrosine residues in JAK2 and the cytoplasmic tail of EPOR ^114, 115^. *Sh2b3* ^−/−^ mice exhibit features of myeloproliferative neoplasms (MPNs) such as splenomegaly and extramedullary hematopoiesis, and inactivating *SH2B3* mutations are associated with myeloproliferative disease in humans ^115^. Genome-wide association studies have identified a hypomorphic *SH2B3* variant associated with elevated hemoglobin and RBC counts ^116, 117^, and suppression of SH2B3 production by RNA interference improved the production of RBCs by *in vitro* differentiation of human CD34 ^+^ cells and embryonic stem cells ^118^.

The EPOR is also negatively regulated at the protein level by several mechanisms ^119, 120^. First, the p85 protein, which facilitates EPOR signaling as a regulatory subunit for PI3K ^121^, also promotes EPOR endocytosis and degradation ^122, 123^. Upon EPO stimulation, the casitas B-lineage lymphoma (CBL) protein ubiquitinates p85 bound to the cytoplasmic domain of EPOR, facilitating interaction with the adaptor protein Epsin-1 to promote endocytosis. Second, prolyl hydroxylase D3 (PHD3)-mediated proline hydroxylation of EPOR stimulates its proteasomal degradation ^124^. Third, iron deficiency reduces the expression of EPOR through interactions with TFR2 and Scribble, a scaffold protein that facilitates EPOR recycling ^125^. This mechanism may explain EPO resistance associated with iron deficiency. Dipeptidylpeptidase (DPP4, CD26) expressed on hematopoietic and stromal cells truncates EPO into inactive fragments, reducing its plasma activity ^126^. These examples illustrate how EPOR signaling is terminated by many proteins acting through multiple mechanisms, most of which are components of a negative feedback loop triggered by EPOR activation.

## Recent insights into EPO–EPOR signaling

**Figure 4.**
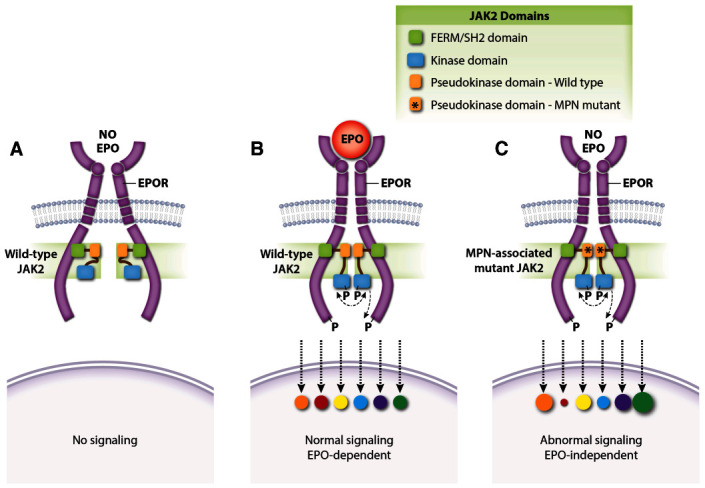
JAK2 regulates dimerization of the EPOR–JAK2 complex. ( **A**) Normal EPOR–JAK2 complexes are inert without EPO. ( **B**) EPO binding to EPOR stabilizes the EPOR–JAK2 complex and triggers downstream signaling by activating JAK2. Colored circles represent normalized activation levels of EPOR signaling targets. ( **C**) Myeloproliferative neoplasm (MPN)-associated mutations in the JAK2 pseudokinase domain, which does not interact directly with EPOR, stabilize dimerization of EPOR–JAK2 complexes and activate JAK2 in the absence of EPO. Mutations in the linker region separating the FERM-SH2 and pseudokinase domains (exon 12) act similarly (not shown). Constitutive activation of the EPOR by MPN-associated mutations causes abnormal downstream signaling relative to that induced by EPO ^142– 146^. EPO, erythropoietin; EPOR, erythropoietin receptor.

The discovery of activating JAK2 mutations in MPNs has fueled the development of ruxolitinib and other JAK2 inhibitors. Ruxolitinib induces clinical responses and improves survival in some patients with MPN but its overall effects and therapeutic index are relatively modest ^127^. New structure–function studies of EPOR and JAK2 may inform the rational design of novel drugs for MPNs. Binding of JAK2 to nascent EPOR in the endoplasmic reticulum facilitates its trafficking to the plasma membrane ^128^. The importance of this protein interaction is freshly reinforced by findings that MPN-associated JAK2 mutants use EPOR as a scaffold for recruiting downstream substrates in order to drive EPO-independent erythrocytosis ^129^. Moreover, changes in the JAK2 pseudokinase domain, which does not interact with EPOR directly, can affect EPOR–JAK2 association ( Figure 4) ^129^. Single-molecule fluorescence microscopy showed that EPO stimulates self-association of EPOR-bound JAK2 through its pseudokinase domain and that MPN-associated *JAK2* mutations strengthen this interaction in the absence of EPO ^130^. Thus, mutant JAK2 proteins drive EPO-independent EPOR signaling by enhancing dimerization of EPOR–JAK2 complexes. Mutant JAK2 also drives MPN by stimulating ligand-independent activation of the thrombopoietin receptor, which is structurally similar to the EPOR. The authors note that MPNs might be treated by drugs which inhibit self-interaction of the JAK2 pseudokinase domain. Although JAK2 binds EPOR through its cytoplasmic box 1, subsequent activation requires another EPOR conserved region, termed the “hydrophobic switch” ^131^. Crystallographic data suggest that this region positions EPOR–JAK2 molecules into a specific conformation that facilitates JAK2 activation ^132^.

**Figure 5.**
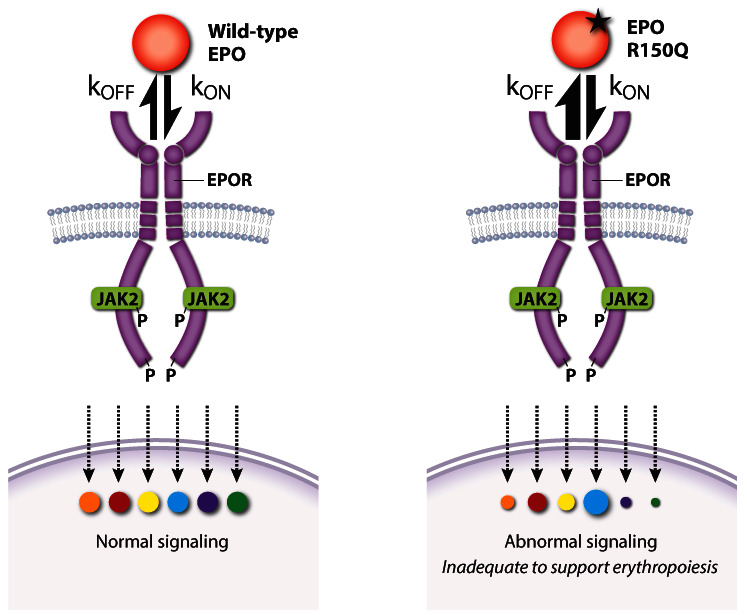
A pathological erythropoietin (EPO) mutant with altered binding kinetics to EPO receptor (EPOR) causes qualitative changes in downstream signaling A homozygous p.R150Q *EPO* mutation was discovered in a patient with severe anemia caused by pure red cell aplasia (Diamond–Blackfan anemia). High levels of the mutant EPO failed to restore erythropoiesis despite an only threefold reduction in its overall affinity for EPOR. Compared with wild-type EPO, the mutant EPO interaction with EPOR was kinetically biased with higher on- and off-rates that altered the activation of specific EPOR effector pathways. k _on_, rate of association; k _off_, rate of dissociation. This figure was created using data from Kim *et al.* 2017 ^133^.

Activated EPOR triggers multiple signaling pathways that interact to specify the activation of different effectors and biological output. Medically relevant insights into this problem were gained by the discovery of a patient with pure red cell aplasia (Diamond–Blackfan anemia) caused by a homozygous *EPO* gene missense mutation (R150Q) ^133^. Although the mutant EPO protein exhibited only threefold reduced steady-state affinity for EPOR, kinetic studies revealed faster on-rate (k _on_) and off-rate (k _off_) ( Figure 5). Abnormally rapid release of the mutant EPO from EPOR was associated with impaired EPOR dimerization and reduced JAK2 activation. Remarkably, alterations in downstream phospho-signaling elicited by the mutant EPO were highly selective. Thus, erythropoietic failure was not caused by complete loss of EPO activity but rather by altered function. This study shows how variability in ligand-induced conformational changes of a cytokine receptor (in this case, EPO on- and off-rates) can selectively alter downstream signaling and biology.

**Figure 6.**
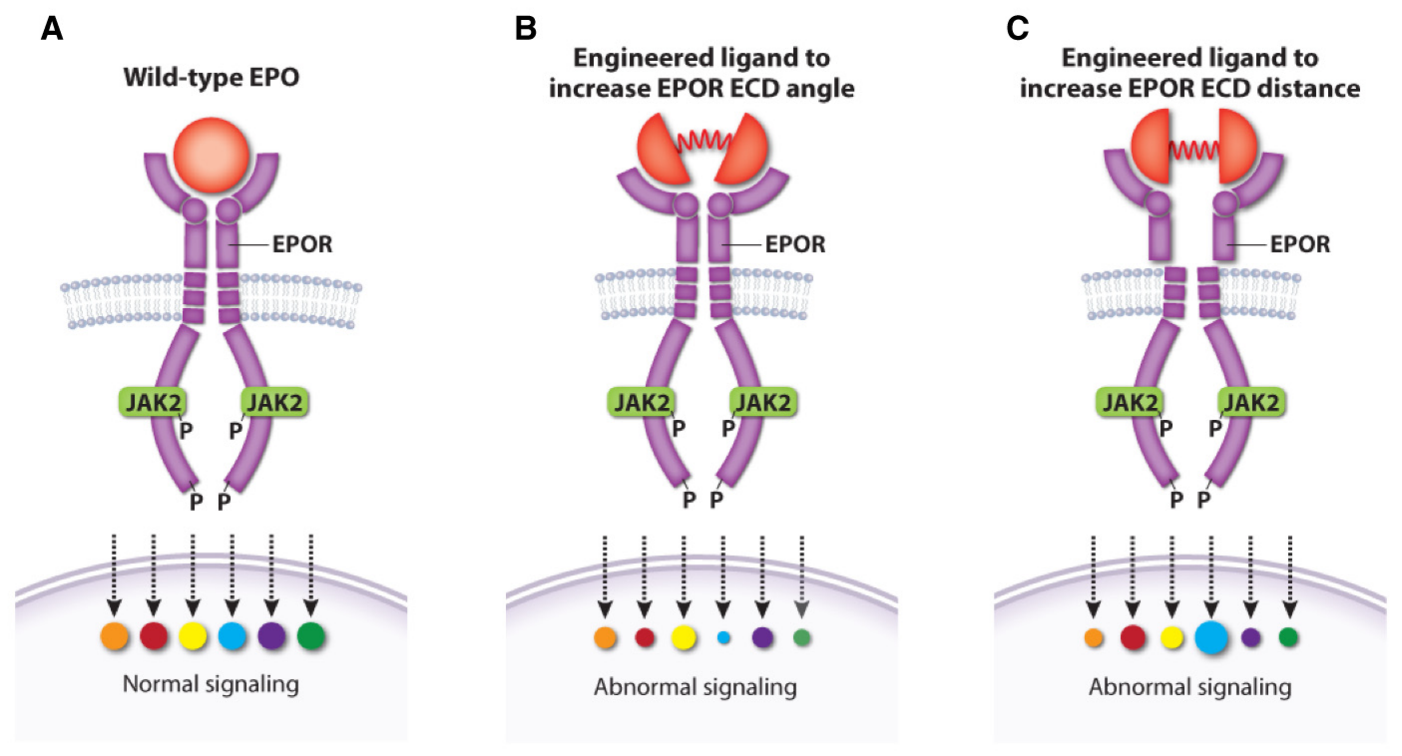
Altered topology of the erythropoietin receptor (EPOR) extracellular domains (ECDs) induced by engineered ligands produces qualitative changes in downstream signaling. ( **A**) Wild-type erythropoietin (EPO) causes normal activation of EPOR signaling targets. Engineered EPOR ligands that modify the angle ( **B**) or distance ( **C**) between EPOR ECDs produce selective alterations in the activation of downstream signaling targets. This figure was created using data from Mohan *et al.*, 2019 ^134^.

Two other studies examined EPO–EPOR structure–function relationships more systematically by designing a series of EPOR ligands that generate different homodimer topologies, resulting in qualitative variation in signaling output ^73, 134^. These findings have potential medical implications. For example, one study showed that different artificial ligands that resulted in different angles and distance between EPOR homodimer subunits generated unique signaling patterns with stage-selective effects on hematopoiesis ( Figure 6) ^134^. The other study ^73^ identified artificial EPOR ligands that can block EPO-independent signaling by the MPN-associated mutation JAK2V617F, which may inform new therapies for MPNs ^134^. Overall, understanding and controlling the signaling output of different EPOR–JAK2 homo-dimer conformations may be used to precisely manipulate hematopoiesis or suppress pathologically active signaling. In this regard, many such studies performed on EPOR are generalizable to other cytokine receptors ^135– 137^.

## Pharmacologic stimulation of erythropoiesis

rhEPO is used to treat anemia associated with a variety of diseases ^13, 138^. The most common indication is anemia of chronic renal failure where rhEPO increases blood hemoglobin levels and improves quality of life ^139^. In general, patients with renal failure have high levels of hepcidin, which limits iron absorption and availability for erythropoiesis ^140, 141^. Thus, rhEPO therapy usually requires administration of intravenous iron ^139^. Although the benefits of rhEPO in renal failure are clear, its overaggressive use is associated with increased rates of arteriovenous fistula thrombosis, venous thromboembolism, congestive heart failure, myocardial infarction, and death ^138, 147– 150^. Similarly, rhEPO use for cancer-related anemia has been associated with reduced survival ^151^. Elevated blood viscosity caused by increased RBC mass probably contributes to these adverse events. Additionally, some adverse effects of rhEPO may result from stimulation of EPOR signaling in non-erythroid tissues or tumor cells or both ^151– 157^, although this point is complicated by technical difficulties in establishing the presence of EPOR in non-erythroid tissues because of non-specific antibody interactions ^158^. Regardless, current guidelines recommend careful titration of rhEPO dosing in patients with renal failure ^139^. A recent study showed that, compared with low-dose intravenous iron sucrose, high-dose intravenous iron sucrose therapy in chronic renal failure resulted in reduced dosage requirements for rhEPO, fewer major adverse cardiovascular events, and lower death rates ^159^.

The routine use of rhEPO in most cancer patients who are undergoing curative chemotherapy should be avoided ^160^. rhEPO remains an important drug for treating anemia associated with myelodysplastic syndrome, although responses are often transient ^161– 163^. From a historical perspective, the first rhEPO (epoetin alfa) was approved for clinical use in 1989 and the longer-acting darbepoetin was approved in 2001. Combined sales reached about $5 billion per year in 2005 and then declined by 40% over the next 6 years as the price of the drugs dropped and the potential adverse effects became recognized ^164^.

The prolyl hydroxylase inhibitors (PHIs), which act by stabilizing HIFα to stimulate endogenous EPO production ( Figure 2), are promising new agents for treating anemia of chronic kidney disease and perhaps other etiologies ^165, 166^. Numerous clinical studies have shown that PHIs are effective for raising hemoglobin levels in subjects with chronic renal failure ^166– 170^. Compared with rhEPO, PHIs offer several potential advantages, including oral administration, improved iron utilization possibly due to suppression of hepcidin, lowering of plasma lipids and cholesterol, and efficacy at relatively low plasma concentrations of endogenous EPO, which may reduce cardiovascular toxicities. Three PHIs are in advanced phase III clinical development, and one was recently approved for clinical use in China ^171^. Although the drugs have been shown to be relatively safe in clinical trials, there are numerous theoretical concerns related to on-target effects given the extensive number of genes and biological pathways that are regulated by the HIF transcription factors. Potential adverse effects include alterations in metabolism, immune response, vascular tone, and angiogenesis. Monitoring for these problems is required in more extended clinical trials pre- and post-marketing.

## Conclusions

EPO and its receptor are essential for the differentiation of CFU-E progenitors into mature RBCs. The complex, multi-layered biochemical pathways that regulate EPO production, signal through EPO engagement of EPOR, and extinguish EPOR signaling are all geared to maintain circulating RBC numbers in a narrow physiological range at steady state and during erythropoietic stress. Examination of these processes over more than 40 years has elucidated fundamental concepts of general biology, defined the mechanisms of human diseases associated with over- or under-production of RBCs, and produced a remarkably useful biological drug to treat some forms of anemia. The development of rhEPO, and more recently PHD inhibitors, arose from basic biological research and represents excellent paradigms for “bench to bedside and back” therapeutic development. Despite the tremendous knowledge gained through extensive studies of EPO and EPOR over many years, the field remains a fruitful area of research, as illustrated by ongoing efforts to better understand the complexities of the PHD–HIF–EPO pathway, structure–function regulation of EPO–EPOR–JAK2 signaling, and mechanisms of human disease caused by germline and somatic alterations in genes tied to EPO biology. In fact, interesting and medically relevant research problems related to EPO are too numerous to cover in a single review. Topics not covered here include the biology of EPOR signaling in non-erythroid tissues and its role in metabolic pathways ^149, 151, 172– 179^. Thus, it is likely that laboratory scientists and clinical researchers who study EPO-related biology and medicine will continue to generate exciting and clinically useful findings for many years to come.
